# Effect of Temperature on the Development and Survival of the Argentine Ant, *Linepithema humile*

**DOI:** 10.1673/031.010.9701

**Published:** 2010-07-08

**Authors:** Silvia Abril, Jordi Oliveras, Crisanto Gómez

**Affiliations:** Department of Environmental Sciences, University of Girona, Montilivi Campus s/n, 17071 Girona, Spain

**Keywords:** brood developmental times, brood survivorship rate, Formicidae, Hymenoptera, invasive species

## Abstract

The influence of temperature on the developmental times and survival of insects can largely determine their distribution. For invasive species, like the Argentine ant, *Linepithema humile* Mayr (Hymenoptera: Formicidae), these data are essential for predicting their potential range based on mechanistic models. In the case of this species, such data are too scarce and incomplete to make accurate predictions based on its physiological needs. This research provides comprehensive new data about brood survival and developmental times at a wide range of temperatures under laboratory conditions. Temperature affected both the complete brood development from egg to adult worker and each of the immature stages separately. The higher the temperature, the shorter the development times. Brood survival from egg to adult was low, with the maximum survival rate being only 16% at 26° C. Temperature also affected survival of each of the immature stages differently: eggs were negatively affected by high temperatures, while larvae were negatively affected by low temperatures, and the survival of pupae was apparently independent of environmental temperature. At 32° C no eggs survived, while at 18° C less than 2% of the eggs hatched into larva. The data from the present study are essential for developing prediction models about the distribution range of this tramp species based on its physiological needs in relation to temperature.

## Introduction

The Argentine ant, *Linepithema humile* Mayr (Hymenoptera: Formicidae), is well known as an invasive ant species (McGlynn 1999). Native to South America (Tsutsui et al. 2001) it has now been introduced into various parts of the world as a result of human commercial activities (Hölldobler and Wilson 1990; Suarez et al. 2001). Its distribution includes areas with Mediterranean-type climates (Passera 1994; Vega and Rust 2001). Its rapid expansion in invaded zones has been facilitated by habitat disturbance (Suarez et al. 1998), but there is increasing evidence of its ability to occupy non-altered habitats (Cole et al. 1992; Holway 1998). In such areas its capacity to negatively affect native ant faunas (Camell et al. 1996; Human and Gordon 1997; Suarez et al. 1998; Holway 1999; Gómez and Oliveras 2003; Sanders et al. 2003) through intense interference and exploitative competition (Holway 1999) has been widely reported. It is also recognized that climatic factors are the key elements that determine its distribution in invaded areas (Roura-Pascual et al. 2004), especially environmental temperature and water availability (Human et al. 1998; Holway et al. 2002; Menke and Holway 2006). Temperature has a strong influence on some reproductive traits of the species: for example, the queens' oviposition rate (Newell 1909; Benois 1973; Abril et al. 2008). *L. humile* brood development rate also seems to be strongly affected by environmental temperature (Newell and Barber 1913; Benois 1973), although data concerning this aspect of its biological cycle are very scarce and incomplete. Such data are essential for predicting, for example, the timing of plague outbreaks or the geographical limits of an insect's distribution (Hartley and Lester 2003). To date, there is little knowledge about how the Argentine ant's biological needs influence its distribution range, since most of the prediction models that have been made are only based on its climatic requirements (Roura-Pascual et al. 2004). Up to now, only few prediction models based on the physiological needs of *L*. *humile* have been made (Hartley and Lester 2003; Krushelnycky et al. 2005), probably due to the poor data available about the influence of abiotic factors on the species' biological cycle.

The relationship between temperature and brood development rates is a useful component in models predicting the areas most suitable for the species to become established in, based on its physiological needs, and as a tool for predicting future changes in its present distribution range as a result of global climate change. However, such models need a considerable amount of data in constant temperature environments to be accurately calibrated (Blank et al. 2000), and the currently available data on the Argentine ant's brood development rates have a lack of replicates for each measured temperature, and daily averages instead of fixed temperatures were used to obtain brood developmental times (Newell and Barber 1913; Benois 1973).

The purpose of this study is to obtain new data about the Argentine ant's brood development times in relation to environmental temperature, not only to improve current knowledge of this species' biology, but also to provide valuable information which will allow the creation of accurate prediction models based on its physiological needs.

## Materials and Methods

### Ant collection and laboratory colonies

*L. humile* used in the study were collected in March 2006 from an invaded natural area situated on the southern edge of the Gavarres Massif near the village of Castell d'Aro (NE Iberian Peninsula) (41° 49′ N, 3° 00′ E).

*L. humile* (14 queens from 14 nests and numerous workers) were collected to create 14 artificial monogynous nests each containing one queen and approximately 300 workers. The nests were incubated at 28° C, which is the optimal temperature for queen oviposition in *L. humile* (Abril et al. 2008). The nests were a variant of those described by Passera et al. (1988), made up of a regular plastic box (180 mm × 115 mm and 35 mm high). The box was fitted with a layer of dry plaster of Paris and was connected laterally to a smaller plastic box (75 mm x 50 mm and 25 mm high) by a cotton wool wick permanently in contact with a piece of cotton soaked in water. To prevent escape, the inner sides of the main plastic box were coated with liquid PTFE (Fluon). The ants were fed daily with a variant of the artificial diet described by Keller et al. (1989). Hashed beef meat was replaced with royal jelly, and the sugar was replaced with honey. The food was not coated with paraffin, but was placed directly on the nest floor. We knew this diet to be very suitable for rearing *L. humile* colonies because it allowed a high fecundity in queens (Abril et al. 2008) and the production of healthy workers and sexuals, both males and queens. The incubation time of these colonies was two weeks. After this period each queen was allowed to lay eggs to obtain the eggs for the study following the same procedure as in Abril et al. (2008).

### Total brood developmental times and survival rates of the worker caste

To study the total immature development period of the worker caste from egg to adult, a total of 100 eggs from the oviposition tests mentioned above were placed in artificial queenless colonies containing approximately 600 workers without brood. In the case of 18° C and 32° C, to obtain reliable data in those extreme conditions, a total of 400 eggs was tested instead of the usual 100.

The artificial colonies were acclimated at ambient temperature in the lab (24–25° C) for ≈ two hours, and then they were kept in environmental chambers at one of seven experimental temperatures (° C ± SD): 18 ± 0.1, 21 ± 0.1, 24 ± 0.1, 26 ± 0.1, 28 ± 0.1, 30 ± 0.1, and 32 ± 0.1. As differences in acclimation could generate differences in the results obtained from each temperature analysed (Jumbam et al. 2008), the same acclimation conditions were used for all treatments.

Observations were carried out daily, and the exact worker development times at each of the seven experimental temperatures were noted. the brood survival rate at each temperature from egg to adult form (including the sexuals) was calculated by means of the data obtained.

### Development times and survival rate of each immature stage

An ant's development from egg to adult form includes three different stages: egg stage, larval stage, and pupal stage. the effect of temperature on development and survival in each of these three stages was studied also.

Due to the small size of the eggs and the difficulty of observing them in the artificial nests (the workers tended to carry them quickly through the nest when observing them under the binocular microscope), additional eggs were incubated in test tube nests instead of in artificial queenless colonies at each of the seven experimental temperatures. The number of eggs tested varied depending on the queen's egg-laying at each temperature and ranged from 105 to 354. The eggs' incubation in the test tube nests was performed without the presence of any workers because previous observations had shown us that there were no differences between the viability of eggs in the care of workers and the viability of eggs without such care, and the observation and individualization of the eggs were easier without workers (when observed under a binocular microscope, they tended to carry the eggs in big masses). The eggs were observed daily from the first day of egg-laying to the appearance of the larva. In this way, more accurate data was obtained by noting the exact number of days from egg to larvae for each egg that hatched. the survival rate of the eggs at each of the seven experimental temperatures was also calculated.

The small size of new-born larvae made it difficult to obtain reliable data about development times in this stage. Therefore, the times were estimated by taking the difference between the total brood development time and the sum of the egg stage and the pupal stage development times. The survival rate of this stage was also estimated from these data.

**Figure 1.**
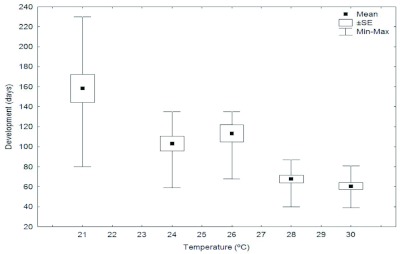
Total developmental times of the Argentine ant, *Linepithema humile, from* egg to adult worker. High quality figures are available online.

The duration of the pupal stage at each of the seven experimental temperatures was measured taking the data from the daily observations of the artificial queenless colonies set up to obtain the total brood development times of the species. Survival rates were also calculated in this stage at each of the experimental temperatures.

## Results

### Total brood development times and survival rates of the worker caste

Temperature substantially affects brood development in *L. humile.* The higher the temperature, the shorter the brood development times (Figure 1). As egg development stopped at 32° C, it can be assumed that this was the upper limiting temperature for total brood development from egg to adult form. In consequence, the larval and pupal development times at this temperature were not analyzed. At 18° C, the survival of the eggs was almost zero: only 1.7% of incubated eggs achieved emergence. For this reason, it was assumed that total development from egg to adult worker at 18° C would be negligible, and therefore, the larval and pupal development times were not analyzed at this temperature.

**Figure 2.**
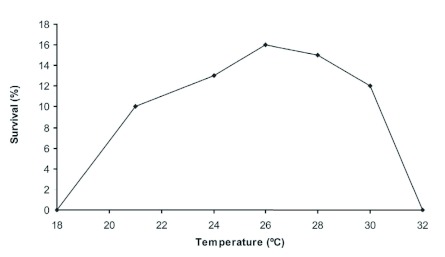
Effect of temperature on brood survival from egg to adult form in the Argentine ant, *Linepithema humile.* High quality figures are available online.

The brood survival rate, expressed in percentage terms, is at its maximum at 26° C and decreases with higher and lower temperatures (Figure 2).

Table 1 summarizes the temperature ranges for brood and worker survival, development, queen oviposition and foraging activity in *L. humile.* We can see that the optimal temperature for the brood survival rate is close to that reported for the queen oviposition, and that these are included within the optimal range of foraging activity, which ranges from 5–15° C to 30–34° C. Above and below this range are the species' lower and upper thresholds for oviposition and foraging activity and the upper and lower lethal limits for survival (Table 1).

### Developmental times and survival rate of each immature stage

**Figure 3.**
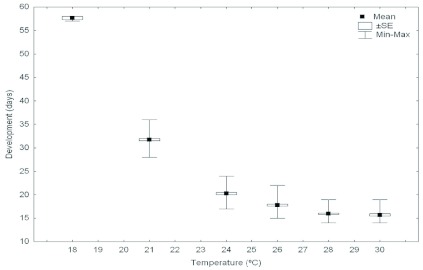
Duration of the egg stage of the Argentine ant, *Linepithema humile,* at different temperatures. High quality figures are available online.

**Figure 4.**
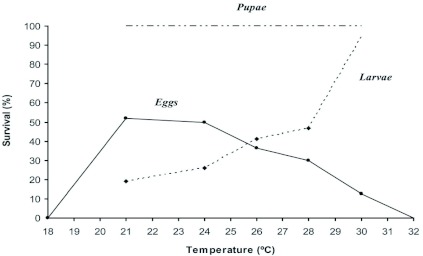
Effect of temperature on the survival of each of the immature stages of the Argentine ant, *Linepithema humile.* High quality figures are available online.

The duration of the egg stage declined from 58 days at 18° C to less than 15 days at 30° C (Figure 3). Eggs at 32° C in the test tube nests (a total of 105) failed to develop because all died in the first two weeks of incubation. At 18° C, virtually none of the incubated eggs in the test tube nests survived: only three of 173 eggs emerged into larva, and the rest died. The eggs placed in the artificial queenless colonies at 32° C (a total of 400) also died in the first two weeks of testing, undoubtedly killed by the extreme temperatures. The incubation range of days decreased as temperature increased (Figure 3). The survival rate of the eggs of this species was negatively affected by high temperatures since only about 13% emerged into larva at 30° C, in comparison with about 52% which emerged into larva at 21° C. Below 21° C the eggs' survival rate was again negatively affected by temperature, since only 1.7% of the eggs achieved the larval stage at 18° C (Figure 4).

**Figure 5.**
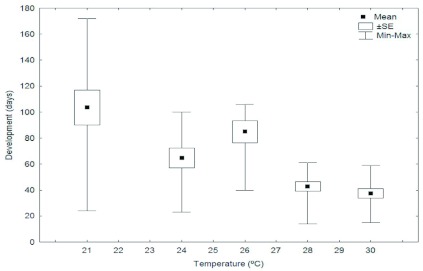
Duration of the larval stage of the Argentine ant, *Linepithema humile,* at different temperatures. High quality figures are available online.

The estimated duration of the larval stage decreased as temperature increased (Figure 5). The range of days to pupal emergence decreased with increasing temperatures. A marked variation in the duration of this stage between samples incubated at the same experimental temperatures (Figure 5) also was observed. The survival rate of this stage seemed to be negatively affected by low temperatures, since only about 19% of the larvae pupated at 21° C, compared with the 94% estimated at 30° C (Figure 4).

**Table 1.**
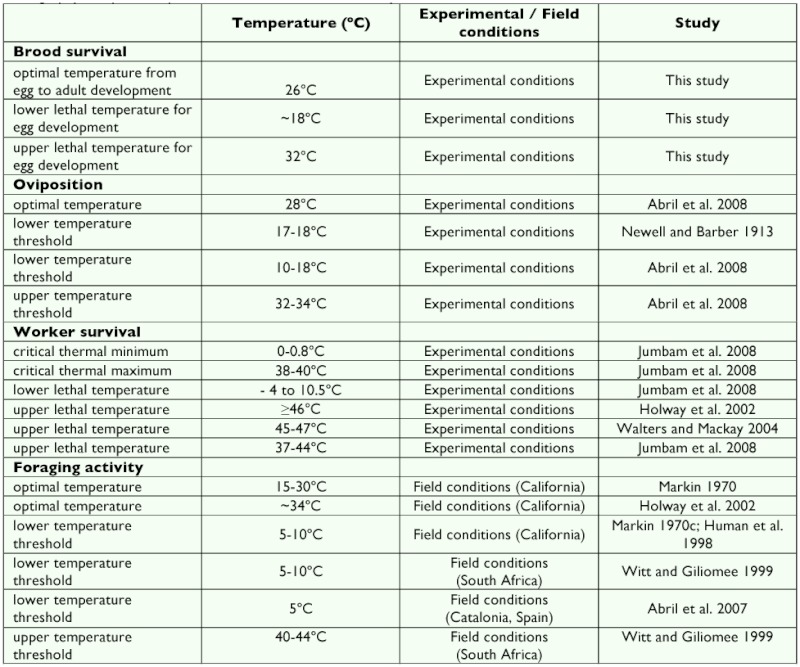
Temperature ranges for brood survival and development reported in the present work with those reported in the biblioeraphy for queen oviposition, worker survival and activity.

**Figure 6.**
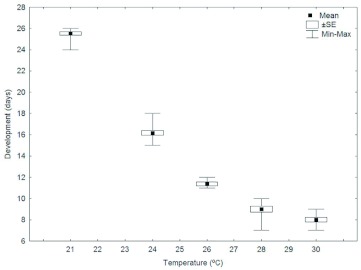
Duration of the pupal stage of the Argentine ant, *Linepithema humile,* at different temperatures. High quality figures are available online.

Within the range tested, the duration of the pupal stage declined from about 25 days at 21° C to about 8 days at 30° C (Figure 6). The range of days of the emergence to adult worker varied little, from one to three days of difference (Figure 6). In contrast to eggs and larvae, the survival of pupae was always 100%), apparently independent of temperature (Figure 4).

## Discussion

The results show that, as was expected, under the range studied (18–32°C), temperature affected the development times of both the complete brood development from egg to adult worker and each of the immature stages in the same manner: the higher the temperature, the shorter the developmental times. This relation was reported by Newell and Barber (1913) almost one hundred years ago. However, our results are generally consistent with those of Newell and Barber (1913) although our data show somewhat longer developmental times than they reported, presumably due to their low number of replicates or differences in experimental conditions.

The effect of environmental temperature on *L. humile* brood development times that was observed in this study has also been observed for other ant species (Porter 1988; Arcila et al. 2002), and in comparison with the results obtained for *L. humile,* the developmental times for some ants like *Solenopsis invicta* (Porter 1988), *Paratrechina fulva* (Arcila et al. 2002) or *Anoplolepis longipes* (Rao and Veeresh 1991) are in general shorter, while *Prenolepis imparis* (Tschinkel 1987) has longer developmental times than *L. humile* at similar conditions of temperature and humidity (27° C, 80% RH).

The upper limit of egg development was 32° C; at this temperature egg survival was zero. The lower temperature limit seemed to be around 18° C; at this temperature the survival of the eggs was practically zero: only three eggs out of 173 emerged into larva. Further research is necessary to know about survival rates and development times for the larval and pupal stages at temperatures above 32°C and lower than 18°C. Because these seem to be the temperatures at which egg development stops, the temperature limit for the survival and development of these two brood stages could be more extreme than those studied in the present work.

In the case of larval developmental times, there is a marked variation between the development times of samples subjected to the same temperature treatment. This could be due to the fact that this form is the only one that is fed by workers (Markin 1970a). This being the case, it would seem that larval developmental times are not only affected by environmental temperature, but also by the food they ingest. This would explain why such a marked difference is only observed in this phase.

Brood survival rate also varies as a function of environmental temperature within the range tested. While eggs were negatively affected as the temperature rose, larvae were negatively affected by low temperatures. This can be explained in the light of the biological cycle of this species in its natural environment. In spring and autumn when the environmental temperature is cool, *L. humile* queens are at their maximum egg-laying period and the maximum egg densities are in the nest at this time, while maximum larvae densities appear at the end of spring when the temperatures are warmer (Markin 1970b; Benois 1973). Therefore, it seems that the *L. humile* biological life cycle, as far as brood development is concerned, is adapted to the physiological temperature needs of each immature form almost during their maximal densities in natural nests. It would allow the maximal survival of the different brood stages, and in consequence, the maximal reproductive success of the colony.

The high survival rate observed in pupae at all the experimental temperatures tested in this study indicates that this phase is the most resistant to temperature variation. This is in accordance with the observations made by Porter (1988), who observed high levels of survival, not related to temperature, in pupae of the ant species *Solenopsis invicta.*

The total brood survival from egg to adult observed in this study was quite low. Only 16 out of 100 eggs reached adult form under the optimal survival temperature of 26° C. In that sense, the percentages of brood survival observed in the present study are in agreement with the ones observed under experimental conditions and similar rearing conditions of temperature and humidity (27° C and 80%, respectively) by Arcila et al. (2002) for the ant species *Paratrechina fulva.* She found a survival of 30% in eggs and of approximately 50%) in larvae, the same percentages obtained in that study at 28° C for *L. humile.* This fact provides confidence in the results obtained. Moreover, studies carried out 50 years ago in Portugal (Silva Dias 1955) on the relationship between brood development times of *L. humile* and environmental temperature, revealed again this low brood survival under experimental conditions. They tried to obtain complementary data to that obtained by Newell & Barber (1913) concerning development from larvae to pupae and from pupae to adult worker. But in the end they were only able to obtain three more pieces of data at different ambient temperatures (larvae to pupae: temperature ≈ 19° C; days of development = 30–31. Pupae to adult worker: mean ambient temperature = 19° C; days of development = 24–26, ≈25° C; 12–16 days and ≈23° C; 16–17 days). This was probably due to the low brood survival rate of this species, at least under experimental conditions, that was observed in the present study.

We believe that brood survival would probably be higher in natural nests due to the thermal gradient present in the nest and the fact that environmental temperature is not constant, but changes throughout the day. The overall likelihood is that this would be used by the workers to incubate the different brood stages at their optimal survival temperature and, in short, assure the maximum reproductive success of the colony. Even though further research is necessary to confirm this, the data given in the present study are very valuable, not only because there are no other studies which offer comprehensive, accurate data about the brood developmental times of *L. humile* at a wide range of fixed temperatures, but also because these data are essential for developing prediction models about the distribution range of this tramp species based on its physiological needs in relation to temperature.
